# Serum Sickness After Cosmetic Botulinum Toxin Type A Injections

**DOI:** 10.1093/asjof/ojag027

**Published:** 2026-02-23

**Authors:** Danielle DeLuca-Pytell, Kathleen Dass, Gabriella Audia, Elena Busuito

## Abstract

Botulinum toxin type A (BoNT-A) is widely used in therapeutic and cosmetic settings for neuromuscular and aesthetic indications. Although generally well tolerated, reports of hypersensitivity reactions—ranging from localized erythema to systemic immune syndromes—underscore its immunological complexity. This case report and literature review demonstrates a novel case of serum sickness following cosmetic Botox® (onabotulinumtoxin-A, Allergan Aesthetics, an AbbVie company, Irvine, CA) administration, categorizes hypersensitivity reactions according to the Gell and Coombs classification (Types I–IV), explores the role of complexing proteins and formulation excipients, and highlights emerging clinical concerns. This will underscore the need for greater vigilance in identifying systemic immune responses to BoNT-A especially within the cosmetic medicine industry.

**Level of Evidence:** 5 (Therapeutic)

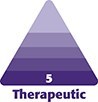

Botox® (onabotulinumtoxin-A, Allergan Aesthetics, an AbbVie company, Irvine, CA) is one of the most commonly used drugs in cosmetic medicine, and neurotoxin treatment is the most common nonsurgical cosmetic procedure in all age groups of both women and men per the most recent Aesthetic Plastic Surgery National Databank statistics.^1^ Although generally considered safe, hypersensitivity reactions—ranging from localized cutaneous findings to systemic immune responses—are increasingly documented. While most reactions are mild, a lack of classification and underreporting of systemic events have led to clinical under-recognition. A recent case of serum sickness following cosmetic Botox® (onabotulinumtoxin-A) injection presents a novel immunologic concern warranting contextualization. A targeted literature review was conducted to further elucidate the prevalence of these hypersensitivity reactions.

## CASE REPORT

**Figure 1.**
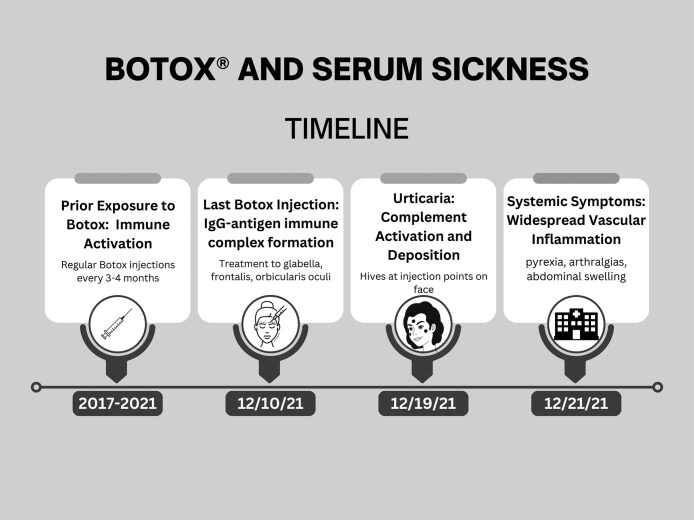
Timeline representing the development of serum sickness for the patient in our case report. Note that exposure to Botox occurred for four years prior to the development of symptoms.

A 46-year-old woman with an unremarkable medical history has had injectable fillers and neurotoxins on multiple occasions in both the United States and Canada. She presents for Botox® (onabotulinumtoxin-A) treatment in Michigan. She describes an interval history of Botox® (onabotulinumtoxin-A) treatment in Canada six months prior and none since. This injection was followed nine days later with urticaria at the injection sites. Two days later (eleven days after injection), she developed pyrexia, symmetric arthralgias, and abdominal swelling and sought care at an Emergency Room (Figure 1).^2^ She was diagnosed with a viral process. When her symptoms persisted, a rheumatology consult was suggested which she declined. Concern about future Botox® (onabotulinumtoxin-A) treatments were raised and treatment was deferred. A consultation with an allergist confirmed serum sickness likely due to albumin in Botox® (onabotulinumtoxin-A). Skin testing was performed with protein-free neurotoxin, incobotulinumtoxin-A (Xeomin®), and no reaction was noted. She has since undergone successful neurotoxin treatment with incobotulinumtoxin-A. Serum sickness did not recur. Written consent was provided by the patient who agreed to the use, analysis, and dissemination of their photos and data.

Review findings confirmed that hypersensitivity reactions to BoNT-A span multiple Gell and Coombs categories (Table 1). Type I reactions (eg, urticaria, angioedema) are typically mediated by gelatin or other stabilizers. Type III immune complex–mediated responses, such as secondary treatment failure and systemic inflammation, are increasingly linked to the presence of complexing proteins. Type IV delayed reactions, including T-cell-mediated pruritus and induration, have been identified. The newly reported case of serum sickness marks the first known occurrence following cosmetic BoNT-A use, confirmed through allergologic workup and successful re-challenge with a protein-free formulation. This reaction fits within the Type III hypersensitivity spectrum and is distinct from previously reported local or pseudoallergic responses. These responses can follow either a single exposure or, more commonly, repeated injections and may be driven by protein components within the formulation or through immune sensitization over time.

**Table 1.** Gell Coombs Classification

Type	Description	Mechanism	Clinical features
I (immediate)	IgE-mediated hypersensitivity	IgE mediated	Anaphylaxis Urticaria Angioedema
II	Antibody-dependent cytotoxicity	An antigen or hapten associated with a cell binds to an antibody	Hemolytic anemia Thrombocytopenia
III	Immune complex disease	Deposition of antigen-antibody complexes in vessels or tissue leading to complement activation and recruitment of neutrophils by interaction of immune complexes with Fc IgG receptors	Serum sickness
IV	Delayed hypersensitivity	T cell mediated	Contact dermatitis Stevens Johnson/toxic epidermal necrolysis Drug reaction with eosinophilia and systemic symptoms

### Type I Hypersensitivity Reactions (IgE-Mediated)

Type I hypersensitivity categorizes IgE-mediated reactions that often occur within minutes to hours after injection. Symptoms include urticaria, pruritus, erythema, and angioedema. Li et al^3^ documented three cases of systemic erythema after facial BoNT-A injection, suggesting early-stage IgE-mediated hypersensitivity. Careta et al^4^ reported a case of urticarial plaques twenty minutes following Botox® (onabotulinumtoxin-A) injection, confirmed by intradermal testing. Positive intradermal testing confirmed an allergic response to bovine gelatin stabilizer in the BoNT-A formulation.

### Type III Hypersensitivity Reactions (Immune Complex-Mediated)

These reactions involve antigen-antibody complexes that activate the complement system, resulting in tissue inflammation and treatment failure. Wanitphakdeedecha et al^5^ linked secondary treatment failure (STF) to elevated anticomplexing protein IgG antibodies in BoNT-A users. Switching to a protein-free formulation restored efficacy. Bian et al^6^ reported a case of a 42-year-old woman who developed delayed-onset swelling and erythema with no therapeutic response to a subsequent BoNT-A formulation, confirming immune complex–mediated secondary resistance.

### Type IV Hypersensitivity Reactions (T-Cell-Mediated)

Delayed hypersensitivity reactions manifest hours to days post-injection and involve sensitized T lymphocytes. Rosenfield et al^7^ described a case in which systemic pruritus and induration appeared 36 hours post-injection, with patch testing confirming T-cell–mediated hypersensitivity. Frevert and Dressler^8^ presented evidence of pseudoallergic responses that mimicked Type I hypersensitivity but lacked prior sensitization, likely involving non-IgE-mediated mechanisms.

### Role of Complexing Proteins and Excipients

Further, complexing proteins such as hemagglutinins, along with excipients like human serum albumin and bovine gelatin, have been implicated in immune activation. Frevert and Dressler^8^ reviewed the immunogenicity of botulinum toxin formulations and showed that traditional BoNT-A preparations containing complexing proteins are more immunogenic than protein-free alternatives like Xeomin®. The inclusion of complexing proteins such as hemagglutinins may function as adjuvants, promoting antibody formation and enhancing the risk of Type III hypersensitivity reactions.^5,8^

### Drug Interactions and Exacerbating Factors

Feng and Liu^9^ reported a case in which a patient concurrently taking cefprozil, a β-lactam antibiotic, developed urticaria-like symptoms following botulinum toxin injection. The reaction was hypothesized to result from hapten-carrier complex formation or mast cell sensitization.

### Epidemiological Data

Nicoletti et al^10^ analyzed 718 adverse events from the EudraVigilance database and found that most hypersensitivity cases involved localized pruritus and swelling. Severe systemic reactions, including dyspnea and angioedema, were rarer but more dangerous, particularly in atopic individuals.

### Clinical Strategies for Mitigating Risk

All patients should be screened for allergy history, including known excipient sensitivities. Skin testing and antibody assays should be considered in those patients with previous reactions or suspected secondary treatment failure. Formulations free of complexing proteins (eg, incobotulinumtoxin-A/Xeomin®) can reduce immune activation and Type III hypersensitivity reactions. Finally, extending intervals between injections and avoiding cumulative high doses can also reduce immune sensitivity over time.

### Delayed and Atypical Hypersensitivity

Several case reports note reactions days after injection, with symptoms persisting beyond standard inflammatory windows, including persistent edema, systemic pruritus, and neurological complaints—some suggesting overlapping autoimmune phenomena.

### Serum Sickness Following Cosmetic Onabotulinumtoxin-A Injection

A 46-year-old woman developed urticaria at injection sites, followed two days later by fever, symmetric joint pain, and abdominal swelling (Figure 1). Symptoms began eleven days post-injection, consistent with classic serum sickness latency. Albumin in the formulation of Botox® (onabotulinumtoxin-A) was identified as the likely antigen. The patient later tolerated protein-free neurotoxin, incobotulinumtoxin-A (Xeomin®), with no recurrence of serum sickness. The reaction is hypothesized to involve IgG-antigen immune complex formation, subsequent complement activation, and widespread vascular inflammation. This case reinforces concerns about BoNT-A formulation components serving as immunogens, even in protein-derived from human sources. This case is the first documented incidence of cosmetic Botox® (onabotulinumtoxin-A)-associated serum sickness, emphasizing the need for high clinical suspicion in patients with systemic symptoms post-cosmetic injection, consideration of formulation switching to non-immunogenic alternatives, and wider awareness of serum sickness in non-core aesthetic medical practices.

## CONCLUSIONS

Hypersensitivity to BoNT-A should be viewed as more diverse than often recognized, with both localized and systemic manifestations mediated through multiple immunologic pathways. The identification of serum sickness following cosmetic onabotulinumtoxin-A administration expands the known adverse event profile and underscores the role of formulation excipients—particularly human serum albumin—in systemic immunogenicity. Clinicians should consider allergy screening, appropriate formulation selection, and diagnostic testing in patients with recurrent or delayed systemic reactions. Discussion of this rare but reported complication is important as BoNT-A use for cosmetic purposes is prevalent in medical spas and non-core physician practices where this complication could be overlooked. This underscores the importance of having close medical supervision for physician extenders as well.
